# Supplementary material for: Population genomic and historical analysis suggests a global invasion by bridgehead processes in Mimulus guttatus
Source: Commun Biol. 2021 Mar 12;4:327. doi: 10.1038/s42003-021-01795-x (PMC7954805; doi:10.1038/s42003-021-01795-x)
Supplement: Supplementary file 1 — Supplementary Information [file 42003_2021_1795_MOESM1_ESM.pdf]

Population genomic and historical analysis suggests a global invasion by bridgehead processes in *Mimulus guttatus*

Mario Vallejo-Marín[1,*], Jannice Friedman[2], Alex D. Twyford[3,4], Olivier Lepais[5], Stefanie M. Ickert-Bond[6], Matthew A. Streisfeld[7], Levi Yant[8], Mark van Kleunen[9,10], Michael C. Rotter[11], Joshua R. Puzey[11]

[1] Biological and Environmental Sciences, University of Stirling. Stirling, Scotland, United Kingdom. FK9 4LA

[2] Biology Department, Queen's University. Kingston, Ontario, Canada. K7L 3N6

[3] School of Biological Sciences, University of Edinburgh. Edinburgh, Scotland, United Kingdom. EH9 3FL

[4] Royal Botanic Garden Edinburgh, 20a Inverleith Row, Edinburgh, United Kingdom. EH3 5LR

[5] INRAE, Univ. Bordeaux, BIOGECO, Cestas, France. F-33612

[6] Herbarium (ALA), University of Alaska Museum of the North, University of Alaska Fairbanks, Fairbanks, Alaska. United States of America. 99775

[7] Institute of Ecology and Evolution, University of Oregon. Eugene, Oregon, United States of America. 97403

[8] Future Food Beacon and School of Life Sciences, University of Nottingham, Nottingham NG7 2RD, United Kingdom

[9] Department of Biology, University of Konstanz. Konstanz, Germany. D-78457

[10] Zhejiang Provincial Key Laboratory of Plant Evolutionary Ecology and Conservation, Taizhou University, Taizhou, China. 318000

[11] Department of Biological Sciences, Northern Arizona University. Flagstaff, Arizona, United States of America. 86011

[12] Biology Department, College of William and Mary. Williamsburg, Virginia, United States of America. 23185

* Author for correspondence: mario.vallejo@stir.ac.uk

Running title: Global invasion of monkeyflowers

Supplementary Methods 1

Introduction history reconstruction by ABC

Our preliminary analyses indicated that introduced *M. guttatus* had a complex origin with multiple introductions in different non-native regions. In order to gain a more detailed understanding of the demographic history of non-native populations, we focussed on the introduction of *M. guttatus* to the United Kingdom, which has been best studied both historically and genetically (Puzey & Vallejo-Marin, 2014; Pantoja *et al.*, 2017). Therefore, we implemented an approximate Bayesian computation approach to determine the most likely *M. guttatus* introduction history in United Kingdom. For this analysis, we used the pruned data set consisting of 1,498 SNPs but included only individuals from the native range or the UK (405 399 individuals). Individuals from the native range were grouped into one of five groups ("populations") delimited by the genetic clustering and phylogenetic tree analysis (see Results section): North (NAM_NNORTH; N=62), South (NAM_SSOUTH; N=42), Coastal (NAM_CCOAST; N=30), Alaska and British Columbia (AKBC; N=70) or Aleutian (ALE; N=45). Six individuals from two populations (SWC and HAM) that formed a separate genetic group in the native range were not included in this analysis. Individuals from United Kingdom were considered to belong to a single population (UK; N=150).

Because all possible scenarios of divergence between the five native populations would have been computationally impossible to test, native population genetic relationships were determined from the phylogenetic tree topology (see Results section). All the simulations assumed that the North population diverged from an ancestral population at time t_4, from which the South population diverged at time t_5. In addition, Costal population diverged from the ancestral population at time t_3 from which Alaska British Columbia population diverged at time t_2 from which Aleutian population diverged at time t_1. The simulated demographic models share this native population divergence history and only differed by the introduction history into UK.

We first considered simple introduction models where the UK population derived from a single native origin at time t_{0a} (models A1 to A5, Supporting Materials File 1). We then simulated UK introduction from a single origin at time t0a followed by a second introduction at time t_{0b} (two-waves introduction models; models B). To reduce the number of simulated models, only models involving the native population that was found to be the most likely introduced in single origin models were considered. This strategy resulted in the definition of 8 different two-waves introduction models (models B1 to B8, Supporting Materials File 1). We then tested more complex introduction models using a similar logic, modelling three (models C1 to C9), four (models D1 to D8) and five (models E1 to E5) waves introduction models integrating the most

likely origins identified in previous sets of models to defined a restricted number of models to compare.

All native populations and the ancestral population were assumed to have constant effective population size (N1 to N6) with a prior distribution bound between 10 to 10000 haploid individuals. This assumption was made to reduce the number of parameters and the model complexity, in absence of clear hypothesis about effective population size variation though time. Native population divergence times were set to wide uniform prior distributions to reflect the uncertainty of native populations history. Divergence time of North and Costal populations from the ancestral population (t_4 and t_3) were sample between 1000 and 15000 generations back in time, divergence time of South population from North population (t_5), AKBC population from Costal population (t_2) and ALE population from AKBC population (t_1) were sampled between 300 and 7500 generations back in time with rules set such as divergence times respect the phylogenetic tree topology (i.e. $t_5<t_4$, $t_2<t_3$ and $t_1<t_2$). The timing of the first introduction was sampled in a prior distribution bounded between 5 and 100 generations back in time. This parameter specification accounts for relatively well-known historical record of first introduction in UK but at the same time reflects the wide range of generation time estimated expected for this clonal, perennial species (set here from 2 to 35 years depending on the populations). Since the first introduction, the UK population effective population size was assumed to increase following a growth rate sampled in a uniform distribution bounded between -0.0005 and -0.5. Growth rate is a compound parameter computed as the $\log(N_{introduced}/N_{now})/t_{0a}$ and is measured backward in time. This prior specification includes different situations from moderate to very strong population growth. Following the first introduction, subsequent introductions were simulated at time t_{0b} to t_{0e} sampled in a uniform prior distribution bounded between 5 and 100 generations with rules set such as these subsequent introductions happened after the original introduction (that is $t_{0b\text{-}e}<t_{0a}$). This specification considers very different cases where a second introduction immediately followed the first introduction (e.g. one generation apart) or happened very recently. In addition, each subsequent introduction was defined by a migration rate (R_1 to R_4) that represents the proportion of haploid individuals that had an immigrant origin in the introduced population at time of the immigration event. These parameters were sampled in uniform prior distribution bounded between 0.001 and 0.999 to account for different situations: large number of immigrants introduced into UK after an original introduction that did not succeed very well (large R value), versus small number of immigrant introduced in an already large introduced population (small R value). Note that given the large prior distribution specifications, different models may have very similar outcome depending on the sampled parameter values during the simulations (e.g., two-introduction wave scenario with very low R vale is very similar to one-

wave introduction scenario) and simpler models are in part included in more complex model in some parameter space.

For each demographic model, we simulated 10,000 genetic datasets consisting of 1435 independent SNP genotypes for 798 haploid individuals distributed following sample size of all six populations in the real dataset using Fastsimcoal2 version 2.6.0.3 (Excoffier *et al.*, 2013) called by ABCtoolbox version 1 (Wegmann *et al.*, 2010). A custom-made bash script was then passed to ABCtoolbox to add missing genotype in the simulated genotypes at an identical amount to the one observed in the real genotypic dataset. Then, ABCtoolbox was set to call arlsumstat program (Excoffier & Lischer, 2010) to compute summary statistics from the simulated genotypes. All within and between populations available statistics for bi-allelic locus were computed (67 summary statistics). In addition, we also computed summary statistics within and between three defined groups of populations (North and South population in one group, Costal, Alaska British Columbia and Aleutian in a second group and UK in a third group) representing an additional set of 29 summary statistics.

Model comparisons were performed iteratively by confronting more and more complex models to simpler models previously found the most likely on precedent models comparison tests (Table 1). In the first round, the introduction models assume a single introduction from one of the five nation populations. Then in round two, we considered two introductions models that necessarily involved the population origin that was found to be the most likely in round one. This allow us to define eight two-waves introduction models: four with the most likely origin in previous round as the first introduction origin followed by a second introduction from one of the four other native population, and four model that assume that the most likely origin in the previous round constitutes the second introduction while the first introduction originated from one of the four other native population (Table 1). We compared the most likely single introduction model and the eight two-waves introduction models. We then considered more complex models, comparing nine three-waves introduction models and the most likely single and two-waves introduction models (Table 1). We subsequently compared models assuming four-waves and five-waves of introduction while still including more simple models in the comparisons (Table 1). Demographic models were compared using a random forest approach implemented in abcrf R package (Pudlo *et al.*, 2016). First, a classification random forest model was built using 1000 trees and a training dataset consisting of the summary statistics computed for the 10,000 simulated genetic datasets for each model. The classification error rate for each model was estimated using an "out-of-bag" procedure to quantify the power of the genetic data given the models and prior distribution specifications to differentiate the different demographic models. Then, the summary statistics computed based on the observed genotypic data were

used to predict the demographic model that best fit the data using a regression forest with 1000 trees. The number of votes for each demographic scenario and the approximation of the posterior probability of the most likely model were reported.

The overall most likely scenario was used to simulate 100,000 genetic datasets using parameters and prior distributions described above to estimate demographic model parameters. A regression random forest model implemented in the R package abcrf was built based on the summary statistics using 1000 trees. The posterior median, 0.05 and 0.95 quantiles of the model parameters were estimated by random forest regression model based on the summary statistics of the observed genotypic composition.

Supplementary References

Excoffier L, Dupanloup I, Huerta-Sanchez E, Sousa VC, Foll M. 2013. Robust demographic inference from genomic and SNP data. *Plos Genetics* **9**(10).

Excoffier L, Lischer HEL. 2010. Arlequin suite ver 3.5: a new series of programs to perform population genetics analyses under Linux and Windows. *Molecular Ecology Resources* **10**(3): 564-567.

Pantoja PO, Simon-Porcar VI, Puzey JR, Vallejo-Marin M. 2017. Genetic variation and clonal diversity in introduced populations of *Mimulus guttatus* assessed by genotyping at 62 single nucleotide polymorphism loci. *Plant Ecology & Diversity* **10**(1): 5-15.

Pudlo P, Marin JM, Estoup A, Cornuet JM, Gautier M, Robert CP. 2016. Reliable ABC model choice via random forests. *Bioinformatics* **32**(6): 859-866.

Puzey J, Vallejo-Marin M. 2014. Genomics of invasion: diversity and selection in introduced populations of monkeyflowers (*Mimulus guttatus*). *Molecular Ecology* **23**(18): 4472-4485.

Wegmann D, Leuenberger C, Neuenschwander S, Excoffier L. 2010. ABCtoolbox: a versatile toolkit for approximate Bayesian computations. *BMC bioinformatics* **11**.

Supplementary Table 1. Posterior estimation of the demographic parameter of model E4.

Demographic parameter	Prior range	Predicted posterior (median and 90% confidence interval)	Posterior mean squared error	Posterior normalized mean absolute error
**t1**	300 - 7500	3186 (1294 - 5861)	914013	0.24
**t2**	300 - 7500	5070 (2998 - 7123)	1027468	0.18
**t3**	1000 - 15000	7051 (2953 - 12188)	5717673	0.36
**t4**	1000 - 15000	7132 (2170 - 13951)	10629192	0.58
**t5**	300 - 7500	1987 (812 - 5279)	829608	0.26
**t0a**	5 - 100	66 (27 - 97)	676	0.52
**t0b**	5 - 100	47 (8 - 89)	723	0.83
**t0c**	5 - 100	43 (8 - 90)	870	0.74
**t0d**	5 - 100	48 (9 - 89)	689	0.69
**t0e**	5 - 100	38 (8 - 88)	812	0.70
**R1**	0.001 – 0.999	0.5 (0.04 - 0.96)	0.08	4.90
**R2**	0.001 – 0.999	0.55 (0.06 - 0.96)	0.07	1.84
**R3**	0.001 – 0.999	0.53 (0.05 - 0.98)	0.08	1.91
**R4**	0.001 – 0.999	0.56 (0.07 - 0.96)	0.06	1.81
**g0**	-0.5 - -0.0005	-0.28 (-0.48 - -0.04)	0.03	0.85
**N0**	10 - 10000	5936 (2171 - 9628)	6623739	0.63
**N1**	10 - 10000	7527 (2520 - 9772)	1358607	0.20
**N2**	10 - 10000	8650 (5851 - 9918)	1915195	1.76
**N3**	10 - 10000	8627 (5707 - 9938)	1972552	0.52
**N4**	10 - 10000	8656 (5124 - 9919)	1850074	0.17
**N5**	10 - 10000	8506 (5310 - 9867)	1661666	0.43
**N6**	10 - 10000	2751 (287 - 7684)	4559228	1.72

t1 : ALE divergence time (in generations) from AKBC; t2 : AKBC divergence time from NAM_C; t3 : NAM_C divergence time from the ancestral population; t4 : NAM_N divergence time from the ancestral population; t5 : NAM_S divergence time from NAM_N; t0a : first introduction time from ALE to UK; t0b : introduction time from NAM_S to UK with migration rate R1 (proportion of haploid individuals that had an immigrant origin in the introduced population at time of the immigration event); t0c : introduction time from NAM_N to UK with migration rate R2; t0d : introduction time from AKBC to UK with migration rate R3; t0e : introduction time from NAM_C to UK with migration rate R4; g0 : UK population growth rate (defined as the log of the ration of the introduced effective population size over current UK effective population size N0, divided by the time of first introduction to UK t0a).

Supplementary Table 2. Number of sites and individuals maintained after filtering and used in each analysis.

Analysis	Number of loci	Number of individuals	Regions	Taxa
**PCA, k-means,**	1,498	474	All regions	*M. guttatus* only
**fastStructure**	1,498	408	All regions (max. 3 individuals per population)	*M. guttatus* only
**IQ-TREE**	8,978 (including invariant sites)	474/521	All regions	*M. guttatus* only/ All taxa
**ABC**	1,435	399 (798 haploid genomes)	UK and native range only	*M. guttatus* only

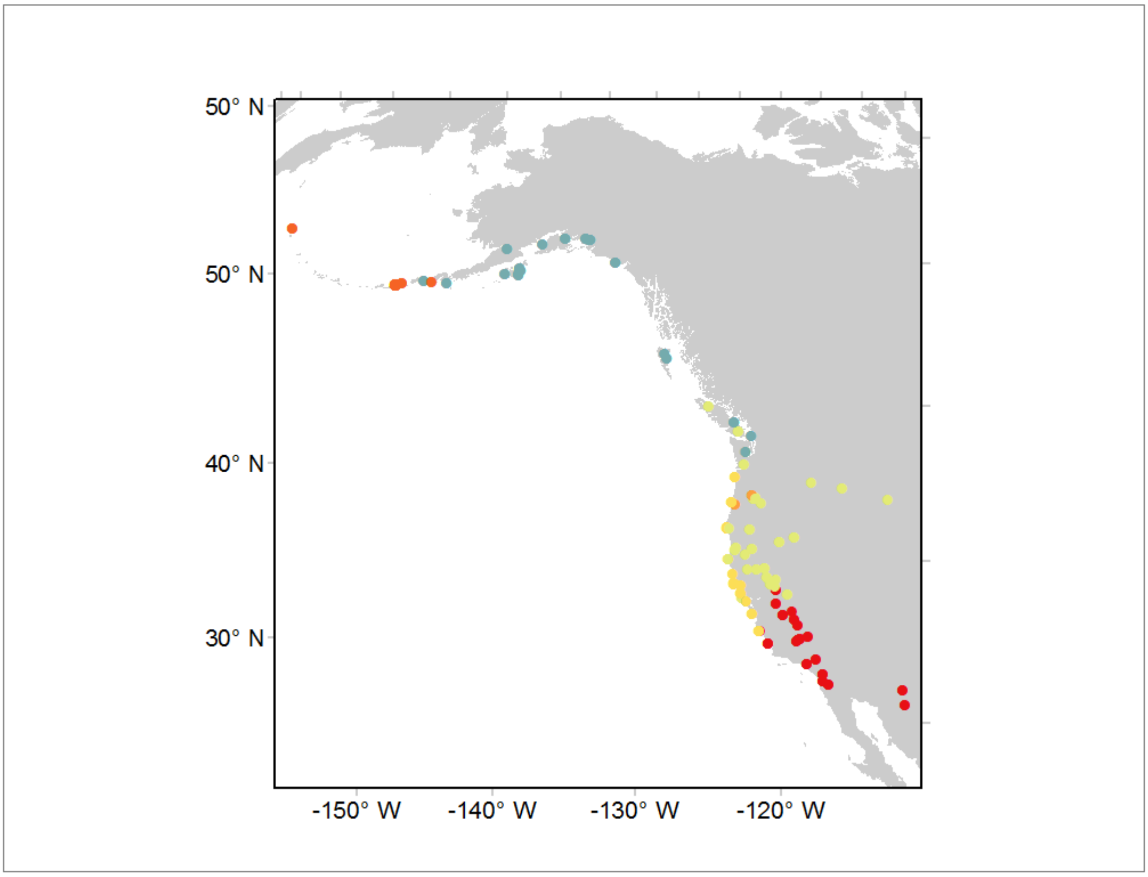

Supplementary Figure 1. Map of North America showing five groups of native *M. guttatus*. Groups were estimated using the global data set by *kmeans* clustering (k=8). Red = South group; yellow = North group; dark yellow = Coastal group; Blue = North Pacific group; orange = Aleutian group.

Supplementary Figure 2. Population genetic structure of native and introduced populations of *Mimulus guttatus* inferred in a Bayesian approach using *fastStructure* (K=2 to K=8). For this analysis, all populations were limited to a maximum of 3 individuals per population. Individuals within geographic regions are arranged by cluster membership. Alaska (native), Western North America (native); ENA = Eastern North America (introduced); GER = Germany (introduced); FO = Faroe Islands (introduced); NZ = New Zealand (introduced); United Kingdom (introduced).

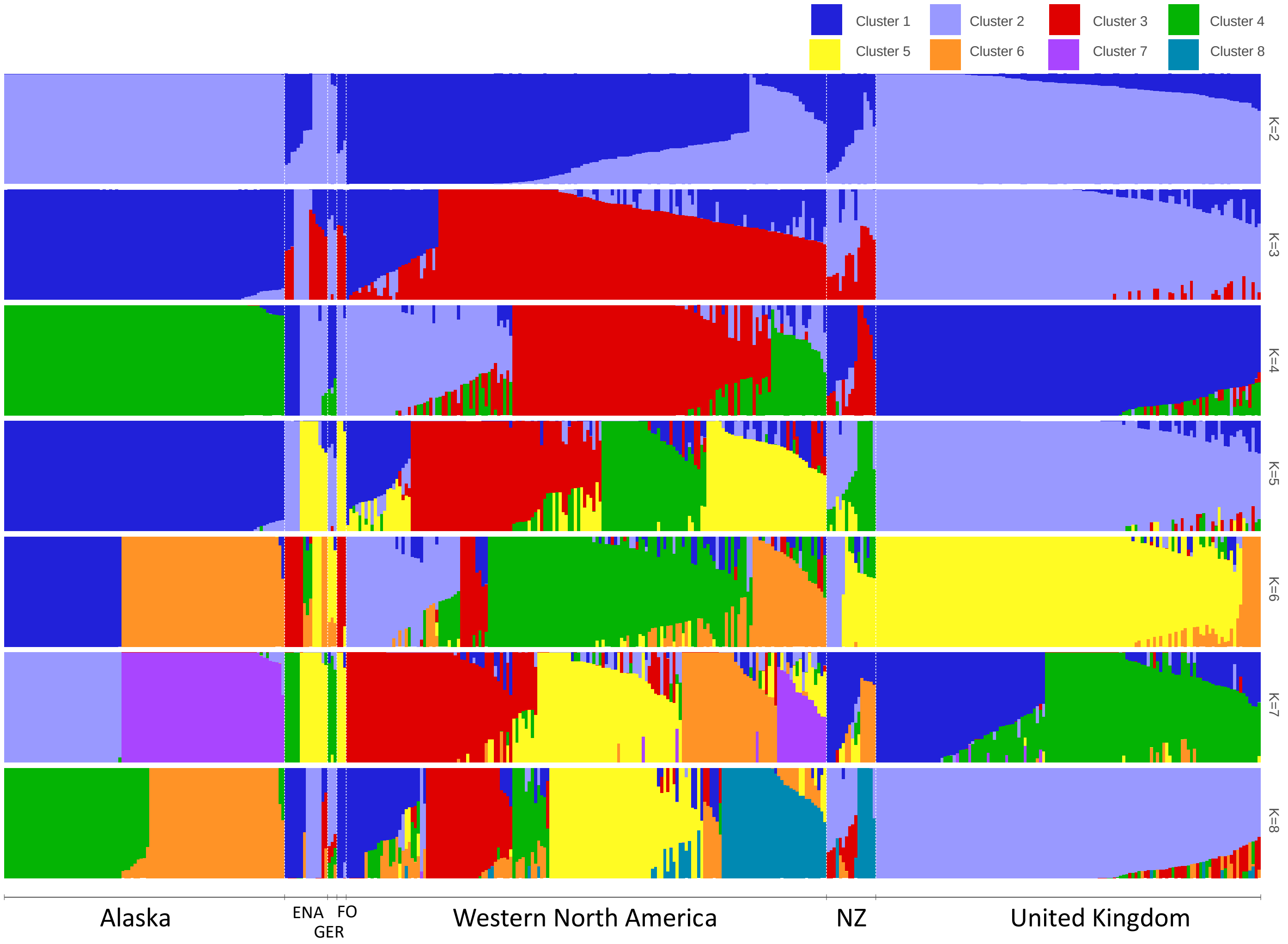

Supplementary Figure 3. Demographic reconstruction of the origin of invasive populations of *Mimulus guttatus* in the United Kingdom using Approximate Bayesian Computation (ABC). The scenario shown here (E4) was selected by hierarchical testing increasingly complex models starting with a single origin of extant UK populations. The model shown here, suggests a first introduction from the Aleutian Islands followed by additional introductions from other parts of the native range of *M. guttatus*.

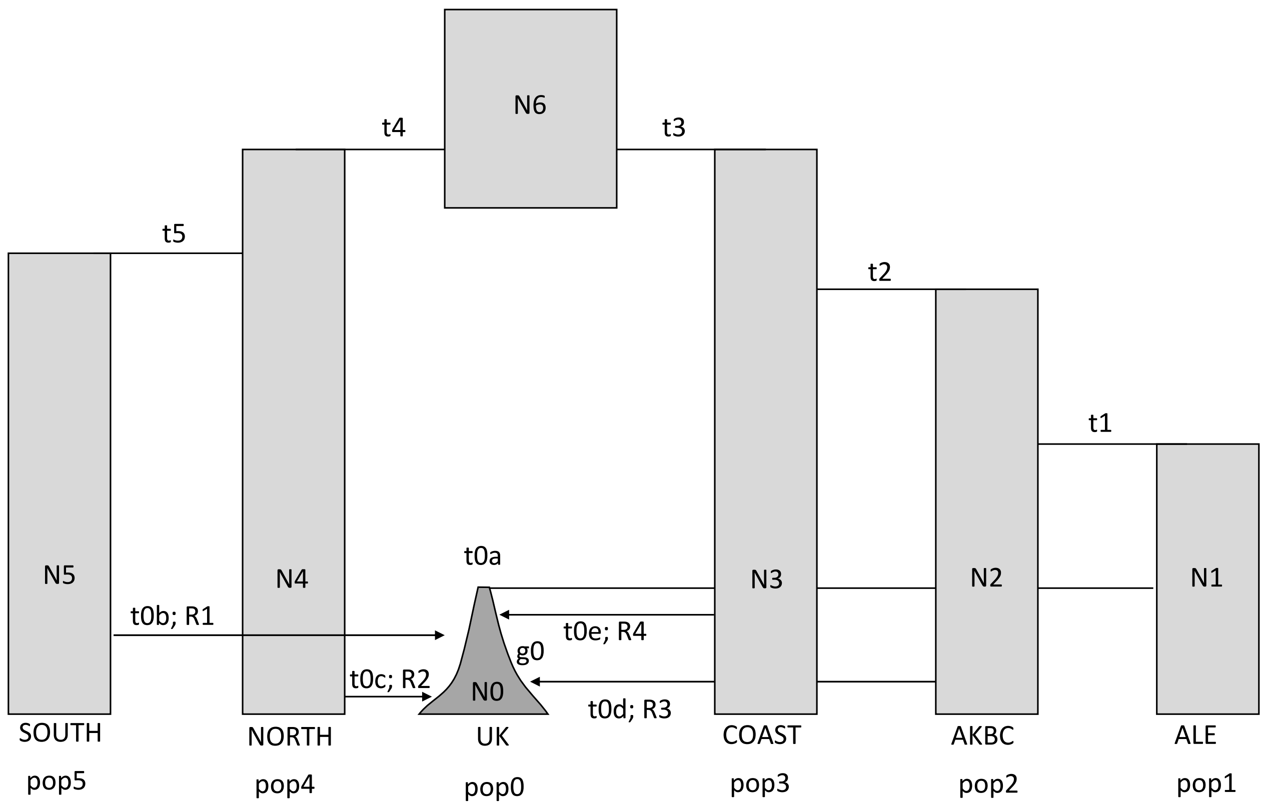

Supplementary Figure 4. Visualisation of the first 200 principal components (cumulative variance explained = 90.9%) projected in two dimensions (V1, V2) using Uniform Manifold Approximation and Projection for Dimension Reduction (UMAP)(McInnes et al., 2018). The Principal Component Analysis (PCA) was conducted on 474 individuals of *Mimulus guttatus* from both native and introduced populations genotyped at 1,498 binary SNP loci. Region: ak = Alaska; nam = western North America; enam = eastern North America; fo = Faroe Islands; uk = United Kingdom; eur = continental Europe (Germany); nz = New Zealand.

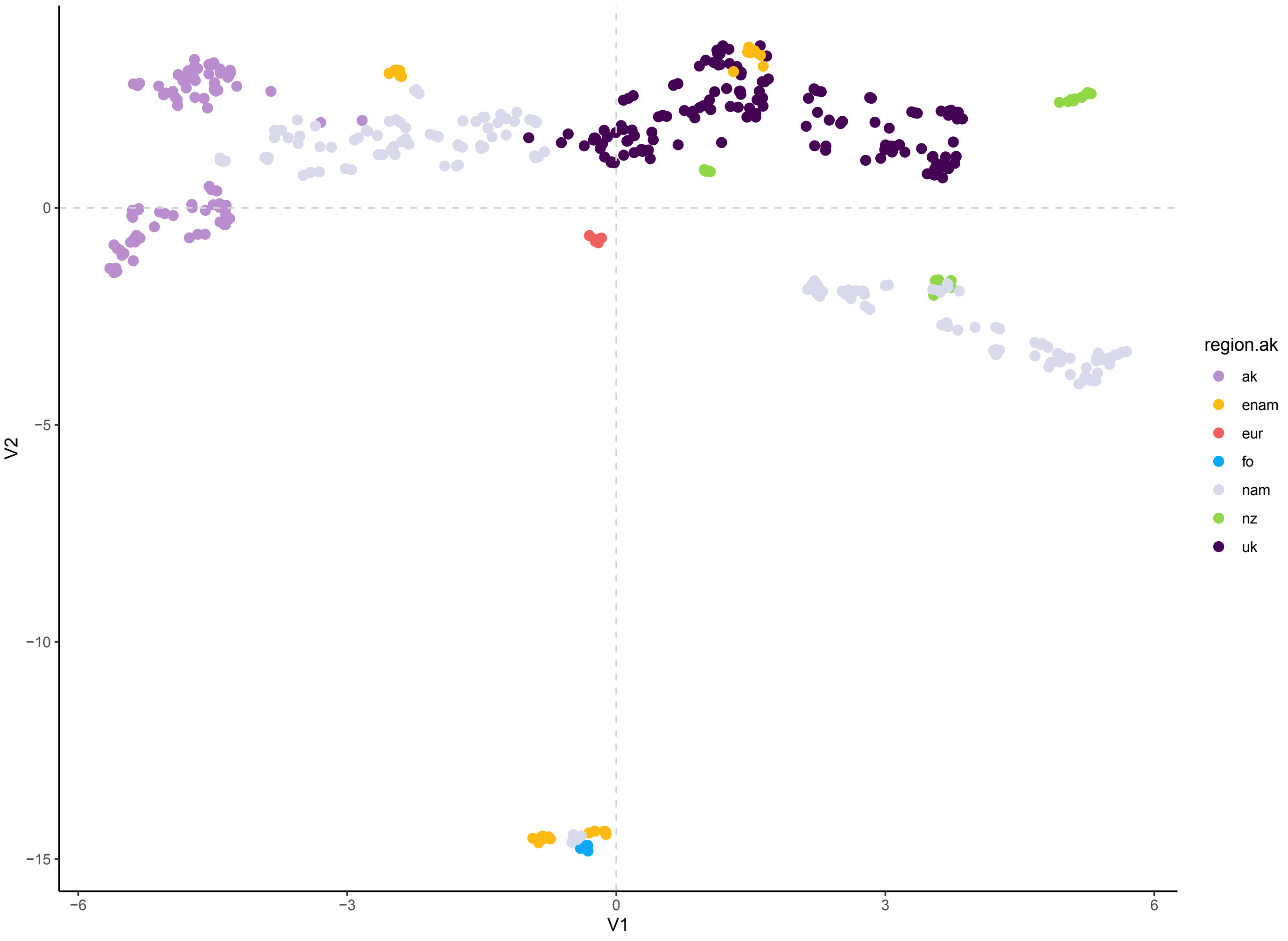

Supplementary Figure 5. Pairwise nucleotide diversity across the genome (π_{GENOME}) for 155 populations of *Mimulus guttatus* in both native and introduced regions. The horizontal line represents the average per region. N = 155 populations; 475 individuals; 20,012 genotyped loci. Region: ak = Alaska; nam = western North America; enam = eastern North America; fo = Faroe Islands; uk = United Kingdom; eur = continental Europe (Germany); nz = New Zealand.

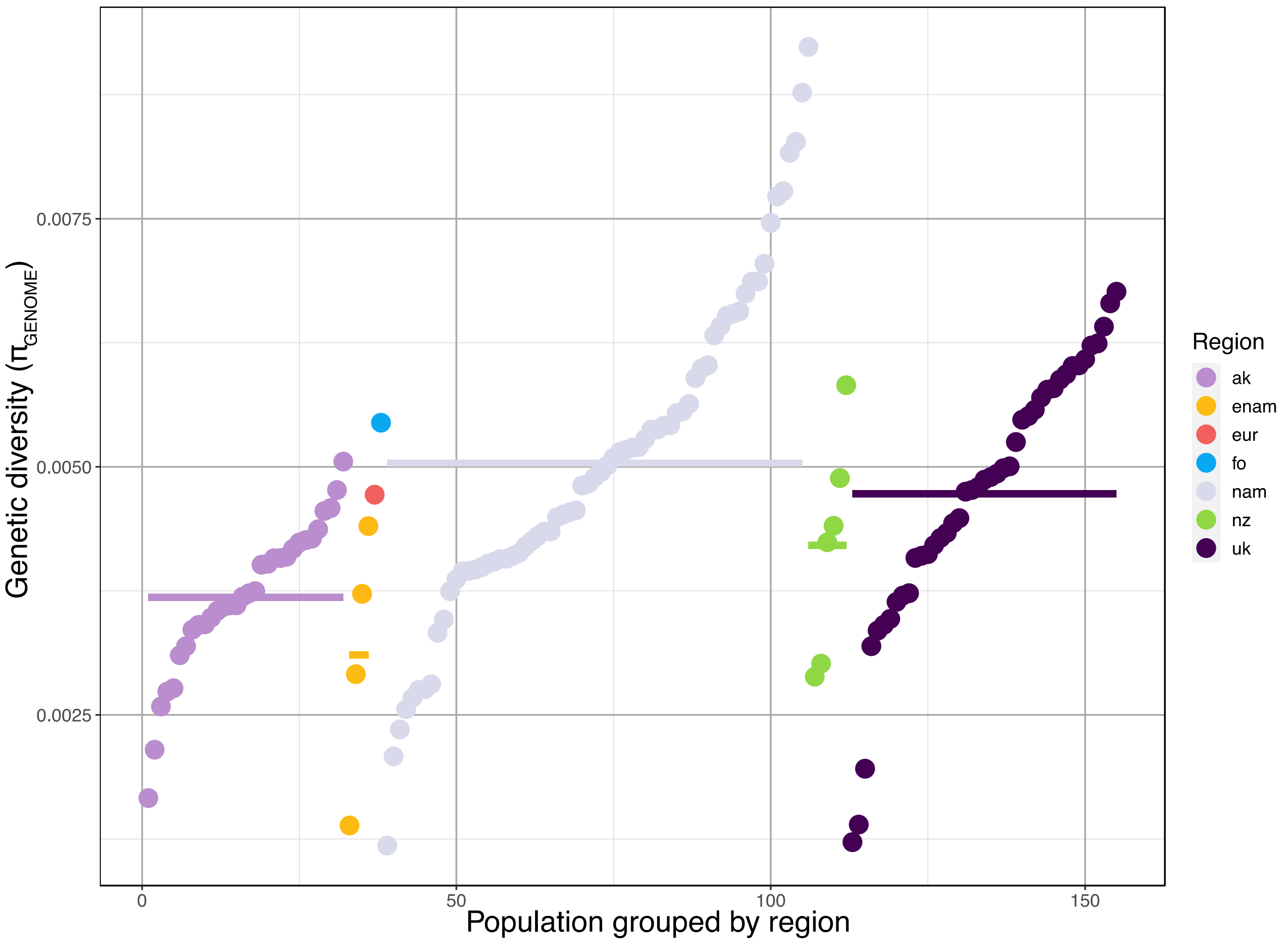

Supplementary Notes 1

Models A: single introduction from one origin

Exemple of demographic scenario – modelA2: introduction from AKBC

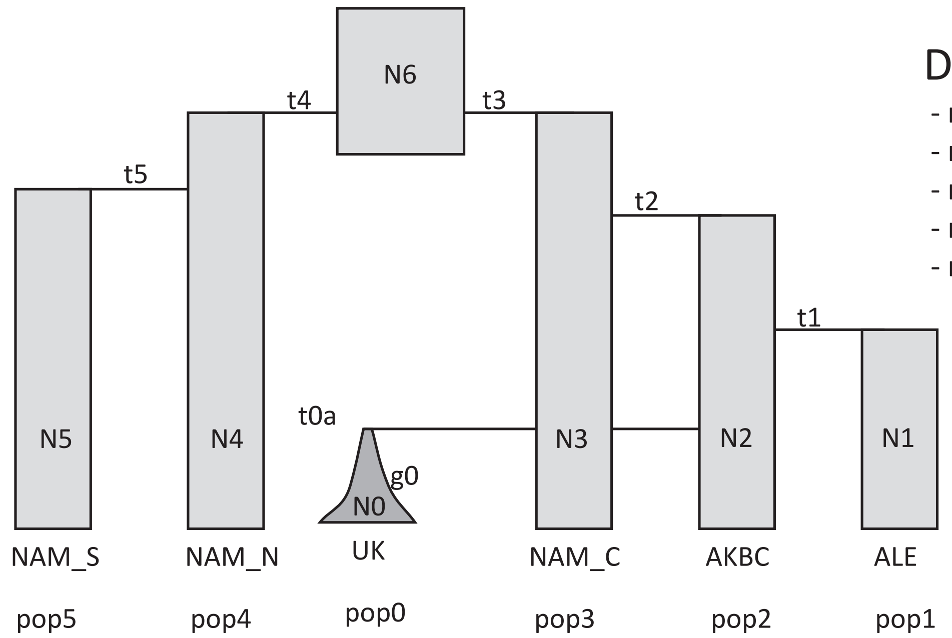

Demographic scenarri simulated:

- model A1: from Aleuthians (ALE)
- model A2: from Alaska / British Columbia (AKBC)
- model A3: from North American Coastal cluster (NAM_C)
- model A4: from North American North cluster (NAM_N)
- model A5: from North American South cluster (NAM_S)

Prior distribution specification

Parameter	Prior distribution	Min	Max	Rules
N0	uniform	10	10000	
N1	uniform	10	10000	
N2	uniform	10	10000	
N3	uniform	10	10000	
N4	uniform	10	10000	
N5	uniform	10	10000	
N6	uniform	10	10000	
t0a	uniform	5	100	t0a < t1
t1	uniform	300	7500	t1 < t2
t2	uniform	300	7500	t2 < t3
t5	uniform	300	7500	t5 < t4
t3	uniform	1000	15000	
t4	uniform	1000	15000	
g0	uniform	-0.5	-0.0005	

Power to discriminate concurent demographic scenarii

Confusion matrix (out-of-bag prior error rate: 2.08%)

Simulated \ Classified	A1	A2	A3	A4	A5	Classification error
A1	**9689**	282	29	0	0	3.1%
A2	315	**9655**	30	0	0	3.5%
A3	20	22	**9957**	0	1	0.4%
A4	0	0	0	**9847**	153	1.5%
A5	0	1	0	187	**9812**	1.9%

Model choice

Votes A1	Votes A2	Votes A3	Votes A4	Votes A5	Best model	Posterior probability
121	**276**	219	206	178	**A2**	89.5%

Models B: 2-waves introduction involving AKBC

Exemple – model B3: introduction from AKBC and then 2nd introduction from NAM_N

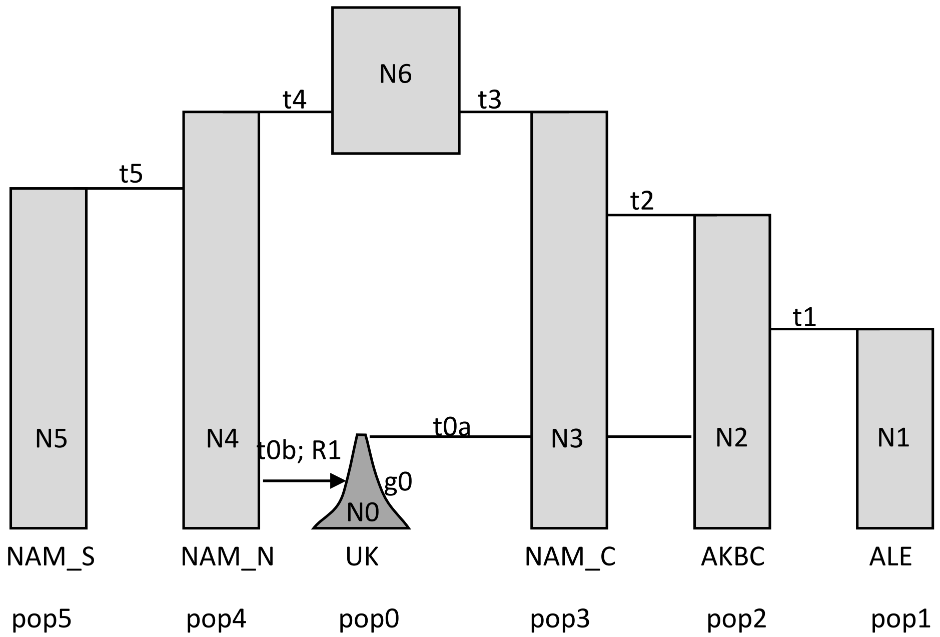

Demographic scenarii simulated

2nd introd. \ 1st introd.	ALE	AKBC	NAM_C	NAM_N	NAM_S
ALE		B1			
AKBC	B5		B6	B7	B8
NAM_C		B2			
NAM_N		B3			
NAM_S		B4			

Prior distribution specification

Parameter	Prior distribution	Min	Max	Rules
N0	uniform	10	10000	
N1	uniform	10	10000	
N2	uniform	10	10000	
N3	uniform	10	10000	
N4	uniform	10	10000	
N5	uniform	10	10000	
N6	uniform	10	10000	
t0b	uniform	5	100	t0b < t0a
t0a	uniform	5	100	t0a < t1
t1	uniform	300	7500	t1 < t2
t2	uniform	300	7500	t2 < t3
t5	uniform	300	7500	t5 < t4
t3	uniform	1000	15000	
t4	uniform	1000	15000	
g0	uniform	-0.5	-0.0005	
R1	uniform	0.001	0.999	

Power to discriminate concurent demographic scenarii

Confusion matrix (out-of-bag prior error rate: 51.57%)

Simulated \ Classified	A2	B1	B2	B3	B4	B5	B6	B7	B8	Classification error
A2	**8161**	617	232	21	29	722	181	21	16	18.4%
B1	**1709**	**4507**	281	37	29	**3221**	177	18	21	54.9%
B2	**1030**	499	**4667**	38	49	413	**3258**	27	19	53.3%
B3	623	270	232	**4270**	659	229	140	**3109**	468	57.3%
B4	618	247	222	607	**4311**	235	139	509	**3112**	56.9%
B5	**1999**	**3271**	211	16	18	**4203**	234	21	27	58.0%
B6	**1311**	321	**3144**	18	37	479	**4623**	41	26	53.8%
B7	705	154	151	**2936**	515	230	184	**4400**	725	56.0%
B8	649	160	157	421	**3008**	219	201	739	**4446**	55.5%

Model choice

Votes A2	Votes B1	Votes B2	Votes B3	Votes B4	Votes B5	Votes B6	Votes B7	Votes B8	Best model	Posterior probability
32	45	78	**237**	172	30	92	183	131	**B3**	48.1%

Models C: 3-waves introduction involving NAM_N and AKBC

Exemple – model C2: introduction from AKBC followed by 2 introductions from NAM_C and NAM_N

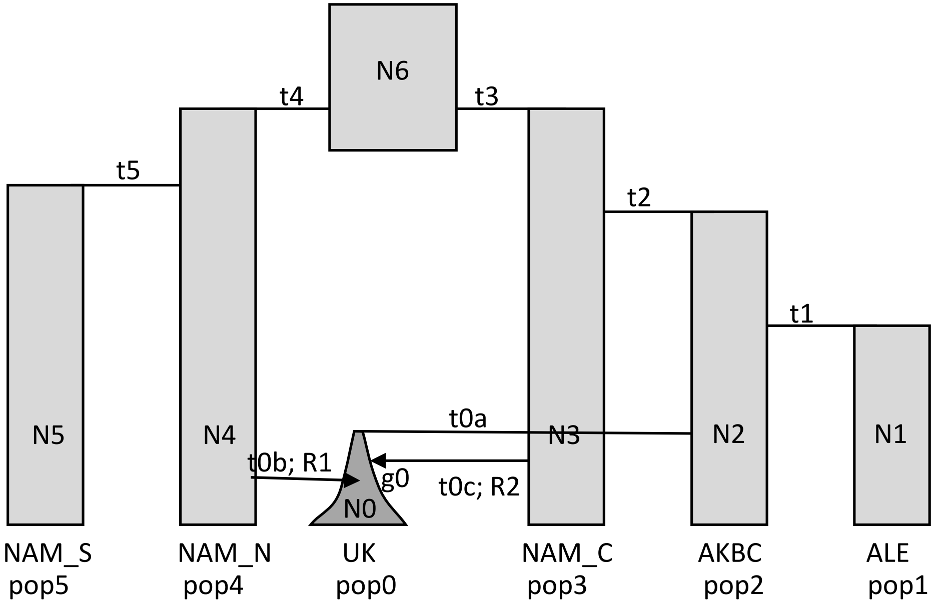

Demographic scenarii simulated

1st introd. / 2nd & 3rd introd.	ALE	AKBC	NAM_C	NAM_N	NAM_S
AKBC & ALE				C4	
AKBC & NAM_C				C5	
AKBC & NAM_N	C7		C8		C9
AKBC & NAM_S				C6	
NAM_N & ALE		C1			
NAM_N & NAM_C		C2			
NAM_N & NAM_S		C3			

Prior distribution specification

Parameter	Prior distribution	Min	Max	Rules
N0	uniform	10	10000	
N1	uniform	10	10000	
N2	uniform	10	10000	
N3	uniform	10	10000	
N4	uniform	10	10000	
N5	uniform	10	10000	
N6	uniform	10	10000	
t0c	uniform	5	100	t0c < t0a
t0b	uniform	5	100	t0b < t0a
t0a	uniform	5	100	t0a < t1
t1	uniform	300	7500	t1 < t2
t2	uniform	300	7500	t2 < t3
t5	uniform	300	7500	t5 < t4
t3	uniform	1000	15000	
t4	uniform	1000	15000	
g0	uniform	-0.5	-0.0005	
R1	uniform	0.001	0.999	
R2	uniform	0.001	0.999	

Power to discriminate concurent demographic scenarii

Confusion matrix (out-of-bag prior error rate: 65.86%)

Simulated \ Classified	A2	B3	C1	C2	C3	C4	C5	C6	C7	C8	C9	Classification error
A2	**8814**	**1032**	18	14	2	62	37	1	10	9	1	11.9%
B3	**2133**	**7260**	54	18	5	348	34	2	131	15	0	27.4%
C1	772	**1200**	**1248**	592	795	**1718**	440	350	**1434**	408	**1043**	87.5%
C2	735	386	607	**1325**	770	576	**1995**	327	417	**1877**	985	86.8%
C3	481	160	580	451	**1718**	569	358	**1767**	402	279	**3235**	82.8%
C4	801	**2138**	984	379	437	**2330**	454	314	**1511**	270	382	76.7%
C5	701	403	416	**1060**	387	626	**3100**	262	284	**2395**	366	69.0%
C6	410	145	502	398	**1303**	598	381	**2576**	316	216	**3155**	74.2%
C7	221	**1690**	**1117**	438	713	**1855**	330	295	**1857**	422	**1062**	81.4%
C8	187	281	426	**1132**	659	322	**2615**	252	407	**2769**	950	72.3%
C9	75	66	339	296	**1519**	259	188	**2116**	352	236	**4554**	54.5%

Model choice

Votes A2	Votes B3	Votes C1	Votes C2	Votes C3	Votes C4	Votes C5	Votes C6	Votes C7	Votes C8	Votes C9	Best model	Posterior probability
28	30	60	**160**	74	98	118	114	96	136	86	**C2**	52.6%

Models D: 4-waves introduction involving NAM_N, NAM_C & AKBC

Exemple – modelD8: introduction from Nam_S followed by 3 introduction from NAM_N, AKBC & NAM_C

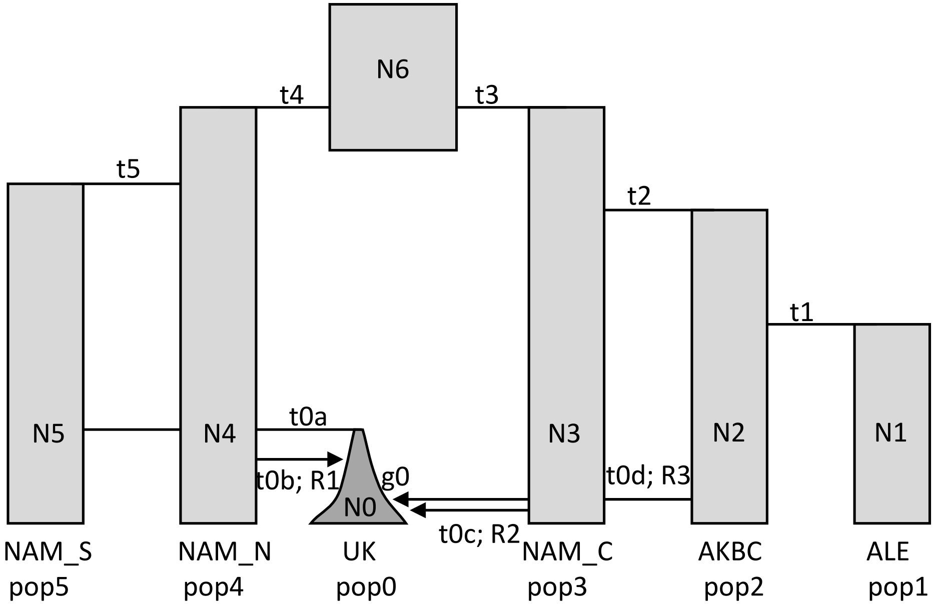

Demographic scenarii simulated

1st introd. / 2nd, 3rd & 4th introd.	ALE	AKBC	NAM_C	NAM_N	NAM_S
NAM_C, AKBC & ALE				D3	
NAM_C, AKBC & NAM_N	D7				D8
NAM_C, AKBC & NAM_S				D4	
NAM_N, AKBC & ALE			D5		
NAM_N, AKBC & NAM_S			D6		
NAM_C, NAM_N & ALE		D1			
NAM_C, NAM_N & NAM_S		D2			

Prior distribution specification

Parameter	Prior distribution	Min	Max	Rules
N0	uniform	10	10000	
N1	uniform	10	10000	
N2	uniform	10	10000	
N3	uniform	10	10000	
N4	uniform	10	10000	
N5	uniform	10	10000	
N6	uniform	10	10000	
t0d	uniform	5	100	t0d < t0a
t0c	uniform	5	100	t0c < t0a
t0b	uniform	5	100	t0b < t0a
t0a	uniform	5	100	t0a < t1
t1	uniform	300	7500	t1 < t2
t2	uniform	300	7500	t2 < t3
t5	uniform	300	7500	t5 < t4
t3	uniform	1000	15000	
t4	uniform	1000	15000	
g0	uniform	-0.5	-0.0005	
R1	uniform	0.001	0.999	
R2	uniform	0.001	0.999	
R3	uniform	0.001	0.999	

Power to discriminate concurent demographic scenarii

Confusion matrix (out-of-bag prior error rate: 69.93%)

Simulated \ Classified	A2	B3	C2	D1	D2	D3	D4	D5	D6	D7	D8	Classification error
A2	**8875**	**1005**	15	15	5	51	3	27	0	4	0	11.3%
B3	**2157**	**7306**	7	18	7	319	5	123	2	55	1	26.9%
C2	199	306	**2841**	843	994	**1179**	498	398	958	626	**1158**	71.6%
D1	549	668	**1756**	**909**	705	**1908**	405	811	762	786	741	90.9%
D2	416	251	**1774**	679	**1048**	891	951	447	**1460**	419	**1664**	89.5%
D3	702	**1913**	**1114**	771	340	**2693**	247	987	150	876	207	73.1%
D4	404	240	**1223**	538	791	937	**1538**	411	**1518**	342	**2058**	84.6%
D5	598	**1664**	**1082**	622	532	**1647**	255	**1349**	718	909	624	86.5%
D6	339	187	895	393	776	455	**1212**	467	**2498**	210	**2568**	75.0%
D7	88	**1238**	**1680**	711	610	**1799**	303	**1011**	654	**1139**	767	88.6%
D8	23	46	**1507**	384	832	361	**1195**	145	**2330**	298	**2879**	71.2%

Model choice

Votes A2	Votes B3	Votes C2	Votes D1	Votes D2	Votes D3	Votes D4	Votes D5	Votes D6	Votes D7	Votes D8	Best model	Posterior probability
22	34	106	116	98	86	122	92	78	110	**136**	**D8**	55.4%

Models E: 5-waves introduction involving NAM_S, NAM_N, NAM_C & AKBC

Exemple – modelE4: introduction from ALE followed by 4 introduction from NAM_N, NAM_S, NAM_C & AKBC

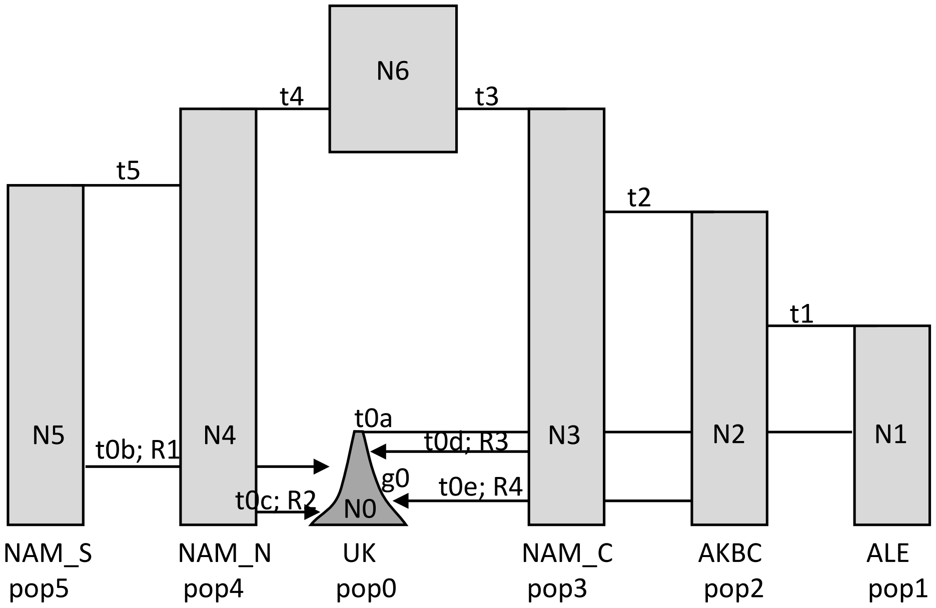

Demographic scenarii simulated

2nd, 3rd, 4th & 5th introd. \ 1st introd.	ALE	AKBC	NAM_C	NAM_N	NAM_S
NAM_S, NAM_N, NAM_C, AKBC	E4				
NAM_S, NAM_N, NAM_C, ALE		E2			
NAM_S, NAM_N, ALE, AKBC			E5		
NAM_S, ALE, NAM_C, AKBC				E3	
ALE, NAM_N, NAM_C, AKBC					E1

Prior distribution specification

Parameter	Prior distribution	Min	Max	Rules
N0	uniform	10	10000	
N1	uniform	10	10000	
N2	uniform	10	10000	
N3	uniform	10	10000	
N4	uniform	10	10000	
N5	uniform	10	10000	
N6	uniform	10	10000	
t0e	uniform	5	100	t0e < t0a
t0d	uniform	5	100	t0d < t0a
t0c	uniform	5	100	t0c < t0a
t0b	uniform	5	100	t0b < t0a
t0a	uniform	5	100	t0a < t1
t1	uniform	300	7500	t1 < t2
t2	uniform	300	7500	t2 < t3
t5	uniform	300	7500	t5 < t4
t3	uniform	1000	15000	
t4	uniform	1000	15000	
g0	uniform	-0.5	-0.0005	
R1	uniform	0.001	0.999	
R2	uniform	0.001	0.999	
R3	uniform	0.001	0.999	
R4	uniform	0.001	0.999	

Power to discriminate concurent demographic scenarii

Confusion matrix (out-of-bag prior error rate: 65.01%)

Simulated \ Classified	A2	B3	C2	D8	E1	E2	E3	E4	E5	Classification error
A2	**8902**	**1043**	24	12	2	9	0	1	7	11.0%
B3	**2151**	**7616**	26	15	4	73	11	25	79	23.8%
C2	210	463	**4844**	**1576**	675	642	534	469	587	51.6%
D8	447	330	**3067**	**1905**	**1039**	662	908	911	731	81.0%
E1	355	336	**2094**	**1342**	**1462**	735	**1323**	**1339**	**1014**	85.4%
E2	400	**1317**	**2161**	**1039**	643	**1473**	756	**1062**	**1149**	85.3%
E3	28	625	**1894**	**1173**	**1277**	857	**1641**	**1438**	**1067**	83.6%
E4	344	**1096**	**1024**	993	**1219**	918	**1313**	**2009**	**1084**	79.9%
E5	435	**1291**	**1491**	777	963	**1170**	**1006**	**1228**	**1639**	83.6%
total								8482		

Model choice

Votes A2	Votes B3	Votes C2	Votes D8	Votes E1	Votes E2	Votes E3	Votes E4	Votes E5	Best model	Posterior probability
46	42	127	106	133	134	120	**151**	141	**E4**	55.1%

Some usefull implications of the results from the confusion matrix:

Given the E4 best model, odds are that the true scenario has is in fact a 4-waves introduction are 10.7% (911/8482), a 3-waves introduction are 5.5% (469/8482), a 2-waves introduction are 0.3% (25/8482) and any 5-waves introduction are 83.4% (7076/8482).

There are 23.7% probability (2009/8482) that the real scenario is the E4 based on all simulated dataset from the prior; our real dataset is more conclusive with 55.1% of probability for scenario E4.

Supplementary Notes 2

Model E4 parameters estimation

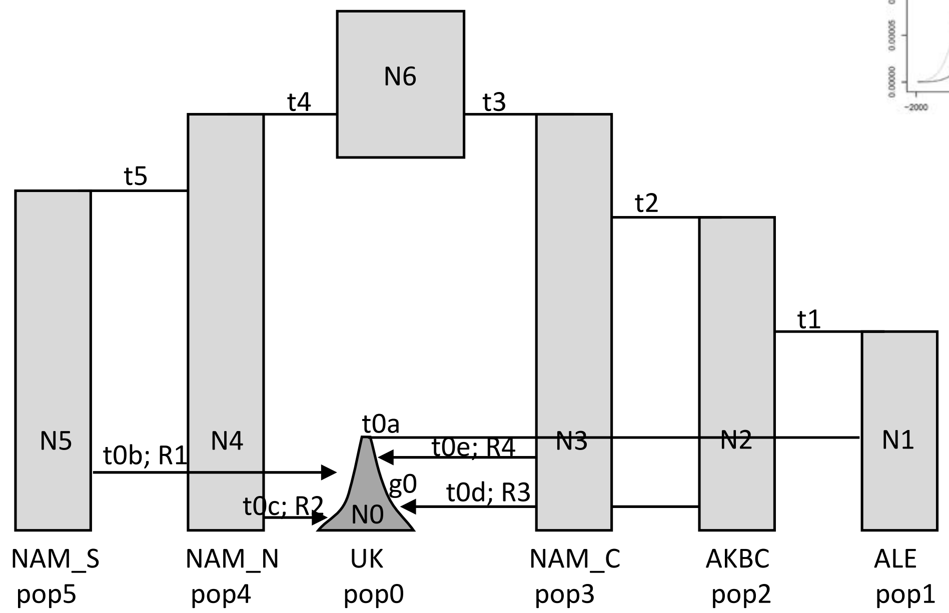

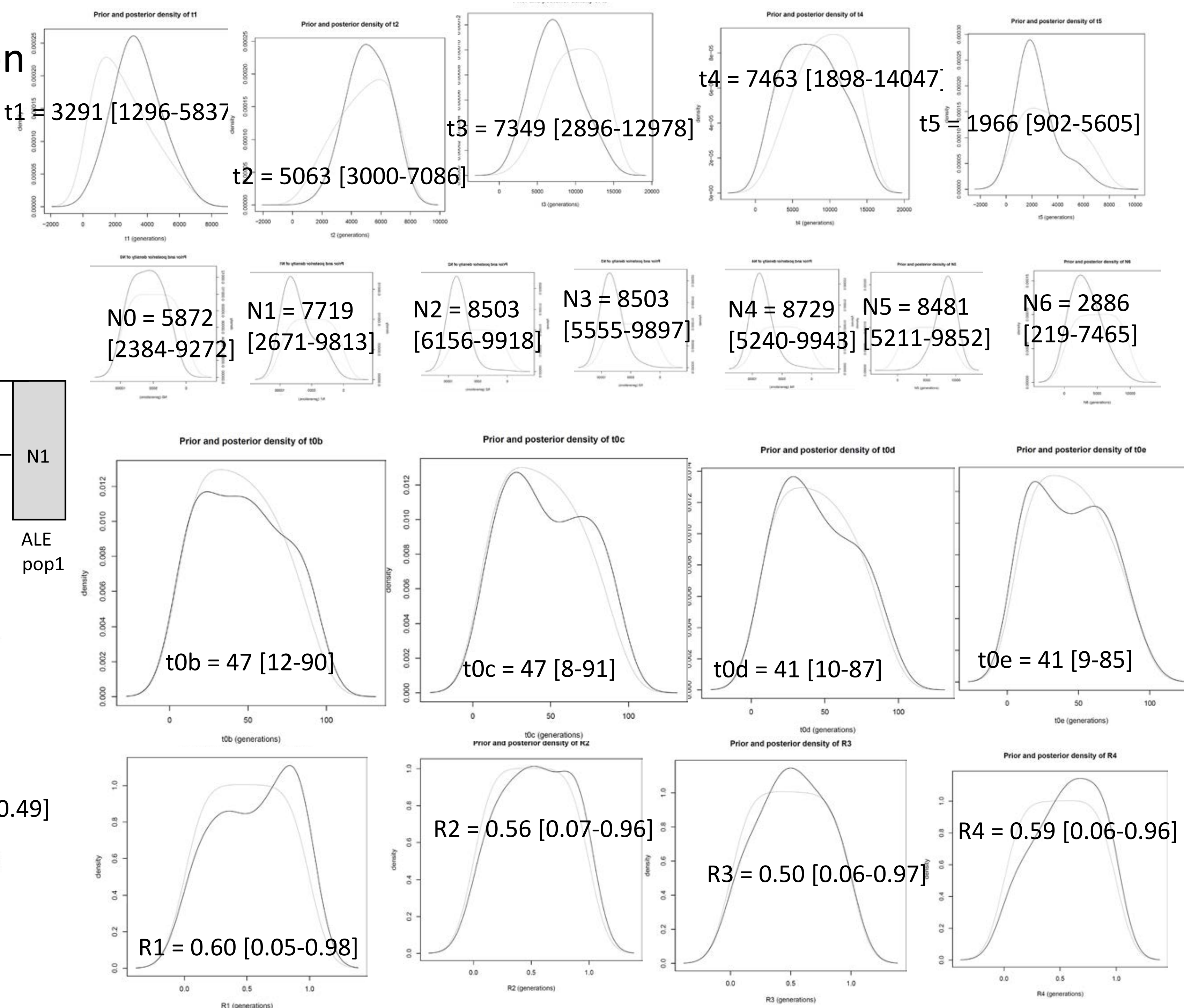

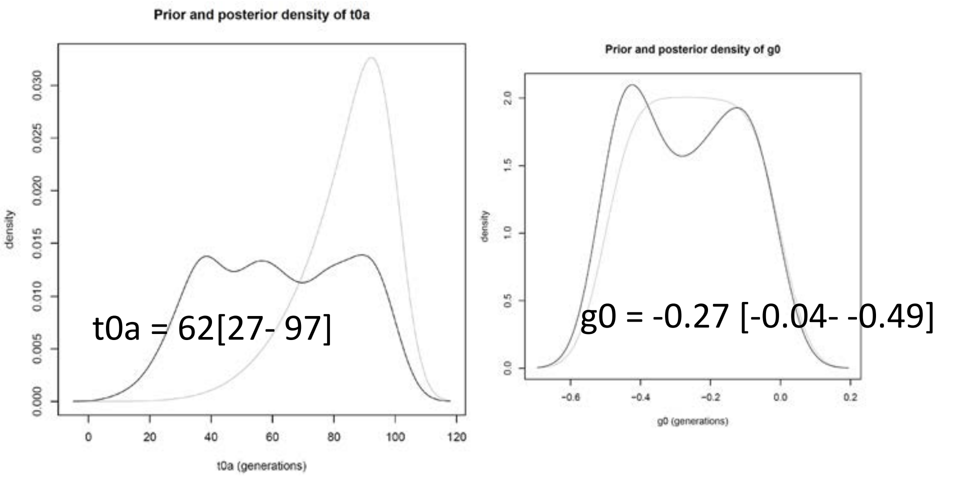

grey line: prior distribution
black line: posterior distribution
