# Supplementary material for: Population genomic and historical analysis suggests a global invasion by bridgehead processes in Mimulus guttatus
Source: Commun Biol. 2021 Mar 12;4:327. doi: 10.1038/s42003-021-01795-x (PMC7954805; doi:10.1038/s42003-021-01795-x)
Supplement: Supplementary file 2 — Supplementary Data 1 [file 42003_2021_1795_MOESM2_ESM.pdf]

Supplementary Data 1. Populations sampled and sequenced. Taxon: gut = *M. guttatus*; gut4x = tetraploid *M. guttatus*, lut = *M. luteus*; rob = *M. x robertsii*; per = *M. peregrinus*, gla = *M. glabratus*. Region: ak = Alaska; nam = western North America; enam = eastern North America; fo = Faroe Islands; uk = United Kingdom; eur = continental Europe (Germany); sam = South America; nz = New Zealand. Life history: A = annual; P = perennial; NA = not available.

Population	Taxon	Region	Latitude	Longitude	Life History	Number of individuals
**00-ATT**	gut	ak	52.89	-172.89	P	3
**16-UGC**	gut	ak	53.82313	-166.434	P	4
**16-ICY**	gut	ak	53.82674	-166.55	P	3
**16-LAU**	gut	ak	53.84003	-166.448	P	4
**16-PTR**	gut	ak	53.84158	-166.521	P	5
**16-ZEN**	gut	ak	53.84306	-166.388	P	3
**16-PTL**	gut	ak	53.8433	-166.396	P	6
**16-PEW**	gut	ak	53.8536	-166.417	P	3
**16-UNA**	gut	ak	53.8698	-166.446	P	4
**16-SLS**	gut	ak	53.8836	-166.454	P	3
**16-EBR**	gut	ak	53.90033	-166.466	P	3
**16-DHS**	gut	ak	53.90448	-166.511	P	4
**16-AMS**	gut	ak	54.13395	-165.777	P	3
**16-FPA**	gut	ak	54.86072	-163.416	P	3
**16-AKC**	gut	ak	55.05603	-162.328	P	3
**16-SDP**	gut	ak	55.32946	-160.502	P	3
**16-OSO**	gut	ak	57.20705	-154.067	P	3
**16-FOS**	gut	ak	57.42559	-152.35	P	3
**16-SZA**	gut	ak	57.45567	-152.45	P	3
**16-KCG**	gut	ak	57.73552	-152.518	P	3
**16-KFC**	gut	ak	57.77976	-152.445	P	3
**16-MFM**	gut	ak	57.79456	-152.595	P	3
**16-ALP**	gut	ak	57.8124	-152.615	P	3
**16-ANT**	gut	ak	57.86179	-152.652	P	3

**02-V142998**	gut	ak	59.02	-155.85	P	3
**03-V153408**	gut	ak	59.793	-141.085	P	3
**16-DCR**	gut	ak	60.02973	-151.683	P	3
**16-CTA**	gut	ak	60.81934	-149.151	P	3
**16-VAT**	gut	ak	61.11486	-145.727	P	3
**16-VPT**	gut	ak	61.11747	-145.777	P	3
**16-VAL**	gut	ak	61.11868	-145.684	P	3
**16-VCC**	gut	ak	61.13814	-146.332	P	4
**15-FLY**	gut	enam	42.7401	-74.9695	NA	9
**15-SNB**	gut	enam	45.69126	-65.82	NA	9
**15-BAS**	gut	enam	46.5484	-65.1101	NA	9
**15-OTO**	gut	enam	46.61653	-89.4282	NA	7
**17-VKL**	gut	eur	47.84194	8.958392	P	9
**13-SKA**	gut	fo	62.19917	-6.85528	P	4
**ALI**	gut	nam	31.2	-111.06	P	2
**CRZ**	gut	nam	32.13283	-111.073	P	3
**KIC**	gut	nam	32.76153	-116.452	A	3
**MUG**	gut	nam	32.9581	-116.914	A	2
**FRC**	gut	nam	33.35229	-116.914	P	3
**SGR**	gut	nam	34.03639	-118.033	P	3
**WMO**	gut	nam	34.28508	-117.374	P	3
**ORO**	gut	nam	35.27333	-120.889	P	5
**FGP**	gut	nam	35.46915	-118.752	P	2
**CDR**	gut	nam	35.50008	-118.694	P	3
**CLC**	gut	nam	35.57347	-118.531	A	3
**SHC**	gut	nam	35.71223	-117.92	P	2
**BCB**	gut	nam	36.06285	-121.592	P	3
**CAN**	gut	nam	36.0689	-121.552	A	3
**EAF**	gut	nam	36.46296	-118.633	P	3
**SCS**	gut	nam	36.78282	-118.9	P	2

**BEL**	gut	nam	37.03983	-119.774	A	3
**LOR**	gut	nam	37.13419	-122.126	A	3
**KAP**	gut	nam	37.27961	-119.105	A	2
**MED**	gut	nam	37.80889	-120.302	A	2
**STB**	gut	nam	37.89139	-122.635	P	2
**MCB**	gut	nam	38.07835	-122.975	NA	3
**FHS**	gut	nam	38.3531	-119.406	P	3
**CVG**	gut	nam	38.3723	-123.055	P	3
**WTB**	gut	nam	38.40525	-123.096	P	2
**CAC**	gut	nam	38.64454	-120.36	A	3
**IHR**	gut	nam	38.81745	-120.374	P	2
**LMC**	gut	nam	38.86398	-123.084	A	3
**OZF**	gut	nam	38.95583	-123.66	P	2
**MIF**	gut	nam	39.00674	-120.748	P	1
**SWB**	gut	nam	39.03611	-123.691	P	3
**NOF**	gut	nam	39.24753	-120.324	A	3
**CAB**	gut	nam	39.35917	-123.817	P	1
**MIY**	gut	nam	39.39442	-121.083	P	3
**WPV**	gut	nam	39.59383	-123.785	P	2
**M2L**	gut	nam	39.8744	-122.634	A	1
**WKY**	gut	nam	39.88002	-121.913	A	3
**NFE**	gut	nam	39.99982	-121.27	P	3
**CMD**	gut	nam	40.40923	-124.392	A-P	1
**BSR**	gut	nam	40.52952	-124.163	P	4
**TAR**	gut	nam	40.85252	-122.866	A	1
**TRR**	gut	nam	41.10861	-123.686	A	3
**LCC**	gut	nam	41.18417	-122.293	P	3
**KLM**	gut	nam	41.27028	-123.606	A	2
**SVH**	gut	nam	41.61556	-120.104	P	3
**BOG**	gut	nam	41.92389	-118.804	P	3
**ASK**	gut	nam	42.38973	-122.476	A	3

**OPB**	gut	nam	42.46389	-124.423	P	3
**RGR**	gut	nam	42.48917	-124.208	A	2
**GEI**	gut	nam	42.49048	-124.419	P	2
**SWC**	gut	nam	43.95944	-123.903	A	3
**BEP**	gut	nam	44.09	-110.72	P	1
**THR**	gut	nam	44.10111	-121.623	P	1
**HEC**	gut	nam	44.13506	-124.123	P	3
**FEV**	gut	nam	44.39988	-122.298	A	3
**IM**	gut	nam	44.40083	-122.151	A	2
**ICS**	gut	nam	44.40722	-122.137	A	2
**TBR**	gut	nam	44.4731	-122.084	P	3
**YJS**	gut	nam	44.95111	-114.584	P	3
**LIN**	gut	nam	45.13583	-123.896	A	2
**CAT**	gut	nam	45.38972	-117.23	P	1
**OSW**	gut	nam	45.76111	-123.983	P	2
**RFA**	gut	nam	46.63083	-123.231	A	3
**HOC**	gut	nam	47.3854	-123.147	A-P	3
**HAM**	gut	nam	47.56518	-123.032	P	3
**AWP**	gut	nam	48.49477	-122.702	A	3
**SKZ**	gut	nam	48.7825	-123.953	A	3
**13-VFC**	gut	nam	49.34516	-124.36	P	4
**NKL**	gut	nam	50.35833	-126.931	A	3
**TSG**	gut	nam	53.41889	-131.916	P	3
**PCL**	gut	nam	53.68889	-132.184	P	3
**12-DUD**	gut	nz	-45.88	170.59	P	6
**12-OTA**	gut	nz	-45.861	170.64	P	2
**12-CHR**	gut	nz	-43.46	172.38	P	2
**13-AMB**	gut	nz	-43.17	172.77	P	5
**13-KAK**	gut	nz	-42.46	173.24	P	5
**13-MOS**	gut	nz	-41.04	172.92	P	5
**14-CRO**	gut	uk	50.16293	-5.29331	P	3

**14-MOO**	gut	uk	50.45142	-4.48601	P	3
**14-TCO**	gut	uk	50.49812	-4.4656	P	4
**14-BOG**	gut	uk	50.79727	-0.69825	P	3
**14-HUN**	gut	uk	50.81071	-0.78888	P	3
**14-DEA**	gut	uk	50.90451	-0.77972	P	3
**10-HOU**	gut	uk	51.09699	-1.5084	P	3
**13-BRA**	gut	uk	52.7681	1.29785	P	3
**10-CER**	gut	uk	53.00598	-3.54927	P	4
**10-WOL**	gut	uk	54.72694	-1.88793	P	3
**12-NEN**	gut	uk	54.80605	-2.37649	P	2
**10-DUM**	gut	uk	55.06738	-3.61335	P	3
**10-AYR**	gut	uk	55.4612	-4.62528	P	3
**10-ETT**	gut	uk	55.53803	-2.87426	P	3
**10-SEL**	gut	uk	55.55088	-2.85027	P	3
**10-COL**	gut	uk	55.65495	-2.24009	P	3
**13-FER**	gut	uk	55.8256	-5.0235	P	3
**14-GLA**	gut	uk	55.8724	-4.28113	P	3
**13-LOM**	gut	uk	56.12926	-4.61327	P	3
**14-STI**	gut	uk	56.13	-3.964	P	3
**14-TIL**	gut	uk	56.1473	-3.74477	P	3
**14-BRI**	gut	uk	56.1557	-3.9512	P	5
**10-ALA**	gut	uk	56.16486	-3.95232	P	3
**11-09-DBL**	gut	uk	56.187	-3.965	P	31
**10-TU6**	gut	uk	56.65369	-3.67581	P	3
**14-LAG**	gut	uk	56.98585	-4.40993	P	3
**10-BAL**	gut	uk	57.23748	-2.06385	P	3
**10-TOM**	gut	uk	57.25499	-3.36777	P	3
**10-ABB**	gut	uk	57.52338	-2.05796	P	3
**14-MAR**	gut	uk	57.5723	-4.4274	P	4
**14-DAL**	gut	uk	57.68261	-4.26526	P	3
**14-CAR**	gut	uk	57.92376	-4.40861	P	3

**15-ELP**	gut	uk	58.06041	-5.02704	P	3
**13-DRN**	gut	uk	58.56763	-4.73794	P	3
**10-DUR**	gut	uk	58.56842	-4.74708	P	3
**13-BKN**	gut	uk	58.5759	-4.76774	P	3
**13-EVI**	gut	uk	59.11226	-3.10809	P	3
**10-BOD**	gut	uk	59.90418	-1.30274	P	3
**10-NIN**	gut	uk	59.97777	-1.30036	P	3
**10-QUA**	gut	uk	60.10456	-1.2268	P	4
**10-MUK**	gut	uk	60.34808	-1.41373	P	3
**10-ESH**	gut	uk	60.48631	-1.61263	P	3
**10-HAM**	gut	uk	60.5034	-1.09931	P	3
**P253**	gut4x	uk	60.10456	-1.2268	P	3
**MLvRC**	lut	sam	-34.2	-70.03	P	1
**10-COL**	lut	uk	55.65495	-2.24009	P	19
**11-LED**	per	uk	55.42366	-3.73493	P	3
**12-WAN**	rob	uk	55.3973	-3.78047	P	3
**15-NAU**	gla	nam	46.08856	-85.4474	P	3
