# Supplementary material for: Population genomic and historical analysis suggests a global invasion by bridgehead processes in Mimulus guttatus
Source: Commun Biol. 2021 Mar 12;4:327. doi: 10.1038/s42003-021-01795-x (PMC7954805; doi:10.1038/s42003-021-01795-x)
Supplement: Supplementary file 5 — Reporting Summary [file 42003_2021_1795_MOESM5_ESM.pdf]

nature research

Corresponding author(s): Mario Vallejo-Marin

Last updated by author(s): Jan 20, 2021

Reporting Summary

Nature Research wishes to improve the reproducibility of the work that we publish. This form provides structure for consistency and transparency in reporting. For further information on Nature Research policies, see our Editorial Policies and the Editorial Policy Checklist.

Statistics

For all statistical analyses, confirm that the following items are present in the figure legend, table legend, main text, or Methods section.

n/a	Confirmed	
☐	☒	The exact sample size (*n*) for each experimental group/condition, given as a discrete number and unit of measurement
☐	☒	A statement on whether measurements were taken from distinct samples or whether the same sample was measured repeatedly
☐	☒	The statistical test(s) used AND whether they are one- or two-sided<br>*Only common tests should be described solely by name; describe more complex techniques in the Methods section.*
☐	☒	A description of all covariates tested
☐	☒	A description of any assumptions or corrections, such as tests of normality and adjustment for multiple comparisons
☐	☒	A full description of the statistical parameters including central tendency (e.g. means) or other basic estimates (e.g. regression coefficient) AND variation (e.g. standard deviation) or associated estimates of uncertainty (e.g. confidence intervals)
☒	☐	For null hypothesis testing, the test statistic (e.g. *F*, *t*, *r*) with confidence intervals, effect sizes, degrees of freedom and *P* value noted<br>*Give P values as exact values whenever suitable.*
☐	☒	For Bayesian analysis, information on the choice of priors and Markov chain Monte Carlo settings
☐	☒	For hierarchical and complex designs, identification of the appropriate level for tests and full reporting of outcomes
☒	☐	Estimates of effect sizes (e.g. Cohen's *d*, Pearson's *r*), indicating how they were calculated

Our web collection on statistics for biologists contains articles on many of the points above.

Software and code

Policy information about availability of computer code

Data collection	*Provide a description of all commercial, open source and custom code used to collect the data in this study, specifying the version used OR state that no software was used.*
Data analysis	Tassel5-GBSv2Pipeline (Glaubitz et al., 2014)<br>IQ-TREE (Nguyen, Schmidt, von Haeseler, & Minh, 2015)<br>ModelFinder (Kalyaanamoorthy, Minh, Wong, von Haeseler, & Jermiin, 2017)<br>R ver. 4.0.0 (R Development Core Team, 2020)<br>fastStructure(Raj, Stephens, & Pritchard, 2014)<br>Fastsimcoal2 version 2.6.0.3 (Excoffier, Dupanloup, Huerta-Sanchez, Sousa, & Foll, 2013)<br>ABCtoolbox version 1 (Wegmann, Leuenberger, Neuenschwander, & Excoffier, 2010)<br>arlsumstat (Excoffier & Lischer, 2010)<br>abcrf (Pudlo et al., 2016)

For manuscripts utilizing custom algorithms or software that are central to the research but not yet described in published literature, software must be made available to editors and reviewers. We strongly encourage code deposition in a community repository (e.g. GitHub). See the Nature Research guidelines for submitting code & software for further information.

Data

Policy information about availability of data

All manuscripts must include a data availability statement. This statement should provide the following information, where applicable:

- Accession codes, unique identifiers, or web links for publicly available datasets
- A list of figures that have associated raw data
- A description of any restrictions on data availability

Genotype data as a VCF file is publicly available at https://datastorre.stir.ac.uk/ (DATAStorre, U. Stirling). Location data of sampled populations is available in the Supplementary Materials (NatGeo_Mimulus.kmz). Herbarium specimens of newly collected material in Alaska is deposited at the ALA herbarium.

Field-specific reporting

Please select the one below that is the best fit for your research. If you are not sure, read the appropriate sections before making your selection.

☐ Life sciences ☐ Behavioural & social sciences ☒ Ecological, evolutionary & environmental sciences

For a reference copy of the document with all sections, see nature.com/documents/nr-reporting-summary-flat.pdf

Ecological, evolutionary & environmental sciences study design

All studies must disclose on these points even when the disclosure is negative.

Study description	Genomic (Genotyping By Sequencing) and demographic (Approximate Bayesian Computation) analysis of native and introduced populations of Mimulus guttatus around the globe.
Research sample	521 plants from 158 native and introduced populations of Mimulus guttatus genotyped at >44,000 loci
Sampling strategy	A small number of individuals were selected per locality to maximise geographic area covered. The same sample strategy has been successfully used in several previous publications in the same model system.
Data collection	Samples collected specifically for this study were collected by Mario Vallejo-Marin, Josh Puzey, Stephanie Ickert-Bond, Jannice Fridman, Martk van Kleunen, Michale Rotter and others.
Timing and spatial scale	Samples collected between 2000 and 2016.
Data exclusions	Data quality control was used as described in the manuscript to exclude samples that failed to meet genotyping standards.
Reproducibility	Failed DNA extractions were repeated as necessary.
Randomization	Samples selected for genotyping were randomly selected for each taxon/population.
Blinding	Three letter code names for each location were used to reduce unconscious bias while analysing the samples.

Did the study involve field work? ☒ Yes ☐ No

Field work, collection and transport

Field conditions	Rain, strong winds, cool temperatures, with the occasional sunny day.
Location	North America, South America, Europe, New Zealand.
Access & import/export	All efforts were made to collect samples in a responsible manner and complying with local and national regulations.
Disturbance	A minimum number of seeds and plants were sampled in each location. The impact on local native populations is negligible as M. guttatus individuals can produce many thousands of seeds per year.

Reporting for specific materials, systems and methods

We require information from authors about some types of materials, experimental systems and methods used in many studies. Here, indicate whether each material, system or method listed is relevant to your study. If you are not sure if a list item applies to your research, read the appropriate section before selecting a response.

Materials & experimental systems

n/a	Involved in the study
☒	☐ Antibodies
☒	☐ Eukaryotic cell lines
☒	☐ Palaeontology and archaeology
☒	☐ Animals and other organisms
☒	☐ Human research participants
☒	☐ Clinical data
☒	☐ Dual use research of concern

Methods

n/a	Involved in the study
☒	☐ ChIP-seq
☒	☐ Flow cytometry
☒	☐ MRI-based neuroimaging
