# Supplementary material for: Population genomic and historical analysis suggests a global invasion by bridgehead processes in Mimulus guttatus
Source: Commun Biol. 2021 Mar 12;4:327. doi: 10.1038/s42003-021-01795-x (PMC7954805; doi:10.1038/s42003-021-01795-x)
Supplement: Supplementary file 6 — Description of Additional Supplementary Files [file 42003_2021_1795_MOESM6_ESM.pdf]

Description of Additional Supplementary Files

File name: Supplementary Data 1

Description: Geolocation (.kmz file) of sampled populations.

File name: Supplementary Data 2

Description: Source data underlying Figures 3 and 4.
